# Personalising care of adults with asthma from Asia: a modified e-Dephi consensus study to inform management tailored to attitude and control profiles

**DOI:** 10.1038/npjpcrm.2016.89

**Published:** 2017-01-05

**Authors:** Alison Chisholm, David B Price, Hilary Pinnock, Tan Tze Lee, Camilo Roa, Sang-Heon Cho, Aileen David-Wang, Gary Wong, Thys van der Molen, Dermot Ryan, Nina Castillo-Carandang, Yee Vern Yong

**Affiliations:** 1 Respiratory Effectiveness Group, Cambridge, UK; 2 Department of Primary Care Respiratory Medicine, University of Aberdeen, Aberdeen, UK; 3 Observational & Pragmatic Research Institute Pte Ltd, Singapore; 4 Asthma UK Centre for Applied Research, Usher Institute of Population Health Sciences and Informatics, University of Edinburgh, Edinburgh, UK; 5 National University Hospital, Singapore, Singapore; 6 Section of Pulmonary Medicine, University of the Philippines-Philippine General Hospital, Manila, Philippines; 7 Seoul National University College of Medicine, Seoul, Korea; 8 Department of Paediatrics, Prince of Wales Hospital, The Chinese University of Hong Kong, Hong Kong, China; 9 Department of General Practice, University Medical Center Groningen, University of Groningen, Groningen, The Netherlands; 10 Department of Clinical Epidemiology, College of Medicine; and Institute of Clinical Epidemiology, National Institutes of Health, University of the Philippines, Manila, Philippines; 11 Discipline of Social & Administrative Pharmacy, School of Pharmaceutical Sciences, Universiti Sains Malaysia, Penang, Malaysia

## Abstract

REALISE Asia—an online questionnaire-based study of Asian asthma patients—identified five patient clusters defined in terms of their control status and attitude towards their asthma (categorised as: ‘Well-adjusted and at least partly controlled’; ‘In denial about symptoms’; ‘Tolerating with poor control’; ‘Adrift and poorly controlled’; ‘Worried with multiple symptoms’). We developed consensus recommendations for tailoring management of these attitudinal–control clusters. An expert panel undertook a three-round electronic Delphi (e-Delphi): Round 1: panellists received descriptions of the attitudinal–control clusters and provided free text recommendations for their assessment and management. Round 2: panellists prioritised Round 1 recommendations and met (or joined a teleconference) to consolidate the recommendations. Round 3: panellists voted and prioritised the remaining recommendations. Consensus was defined as Round 3 recommendations endorsed by >50% of panellists. Highest priority recommendations were those receiving the highest score. The multidisciplinary panellists (9 clinicians, 1 pharmacist and 1 health social scientist; 7 from Asia) identified consensus recommendations for all clusters. Recommended pharmacological (e.g., step-up/down; self-management; simplified regimen) and non-pharmacological approaches (e.g., trigger management, education, social support; inhaler technique) varied substantially according to each cluster’s attitude to asthma and associated psychosocial drivers of behaviour. The attitudinal–control clusters defined by REALISE Asia resonated with the international panel. Consensus was reached on appropriate tailored management approaches for all clusters. Summarised and incorporated into a structured management pathway, these recommendations could facilitate personalised care. Generalisability of these patient clusters should be assessed in other socio-economic, cultural and literacy groups and nationalities in Asia.

## Introduction

The success of treatment in any chronic disease is highly dependent on patient behaviour, their attitude towards the prescribed management approach and subsequent adherence to prescribed treatment regimens. In asthma, successful management of symptoms includes practical trigger avoidance and implementation of (evidence-based) therapy to maximise its potential for benefit and minimise the risk of harm. Yet large, multi-national, population-based studies suggest that only a minority of the 17.5 million of people with asthma in Asia (and over 300 million people globally)^1^ actually achieve good control.^1–4^ The cost of poor control is high, both in terms of patients’ quality of life^5,6^ and their demand on healthcare resources,^7–10^ particularly in Asia where equitable access to affordable, quality care continues to be a challenge. It is therefore important to identify the attitudinal determinants of asthma control, especially potentially modifiable factors, and to intervene with affordable, targeted approaches.^11^

### REALISE Asia

The REcognise Asthma and LInk to Symptoms and Experience (REALISE) Asia Study^12^ assessed patients’ and professionals' perceptions of asthma control and patients’ attitudes towards their treatment across eight participating countries in Asia: People’s Republic of China (30%), Hong Kong (8%), Indonesia (7%), Korea (20%), Malaysia (6%), Philippines (6%), Singapore (8%) and Taiwan (12%). Adults (*n*=2,467) aged 18–50 years receiving treatment for physician-diagnosed asthma were randomly sampled from established (self-reported asthma) patient panels. They were asked (via an online survey) about their asthma symptoms, exacerbations and treatment type, views and perceptions of asthma control, attitudes towards asthma management, and sources of asthma information. Specialist and generalist physicians (*n*=1074) completed a face-to-face and/or online survey exploring their percpetions of the impact of asthma and the way the disease is managed in their country; more details of the survey scope are detailed in the accompanying online supplement.

Echoing findings from North America and Europe,^13–20^ the questionnaire revealed a mismatch in the proportion of patients who believed they had well-controlled asthma (89%), when asked a generic question about ‘control’, compared with the proportion who achieved guideline-defined control status (18%), assessed by responses to specific symptom questions.^12^ Similarly, physicians over-estimated the proportion of their patients who had guideline-defined control (53%). The study also revealed a common misperception (in two-thirds of patients) that ‘control’ related to the ability to manage the acute symptoms of disease rather than preventing symptoms and exacerbations. This was reflected in the finding that over one-third of respondents admitted to ignoring doctors’ instructions on how and when to use prescribed treatments and almost three-quarters believed they could manage their asthma without the help of a doctor. These findings illustrate the important role that asthma patients’ attitudes and perceptions of their condition can have in their receptivity to, and the acceptance and implementation of asthma advice from healthcare professionals.

### Building on the REALISE Asia findings—patient segmentation

**Figure 1:**
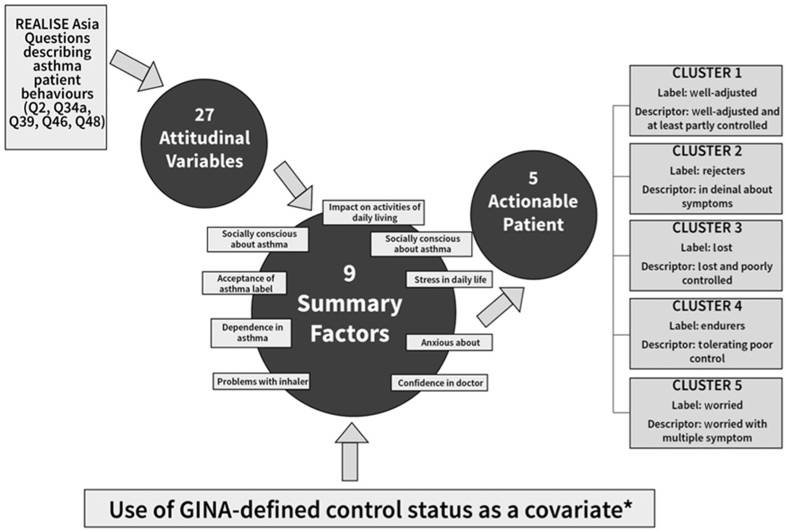
Schematic illustration of the cluster analysis approach used to identify the five attitudinal–control patient clusters within the REALISE Asia population.^22^ *Being a covariate meant that the GINA-defined status had an effect added to or subtracted from the factors used for segmentation, so GINA-defined status did not have an effect in itself but modified the way the other measures affected segment allocation. Thus, segmentation model performance was improved when GINA-defined control status is taken into consideration during regression analysis**.**

**Figure 2:**
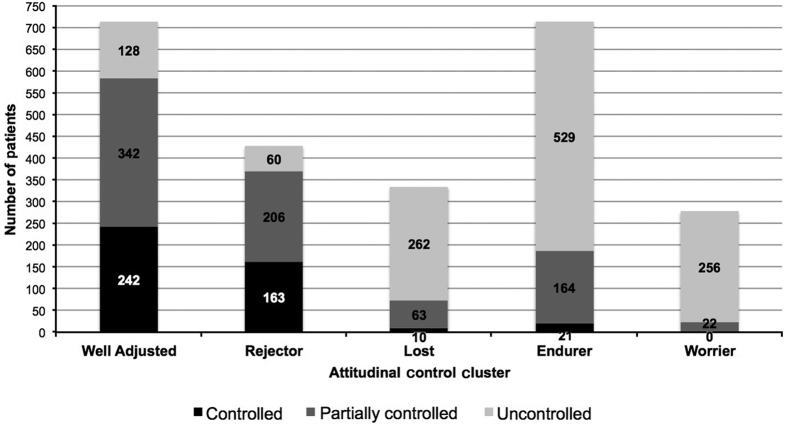
Distribution of REALISE Asia patients across Attitudinal–Control Cluster, categorised by GINA control profile.^22^

Recognising the importance of patient attitudes in achieving a positive patient–physician relationship and collaborative approach to disease management, the REALISE Asia investigators used a two-step approach to segment questionnaire responses and characterise patients in terms of these attitudes (Figure 1). Factor analysis was used to identify nine summary attitudinal factors and cluster analysis to define five attitudinal groupings.^21,22^ Patients in each cluster shared distinct attitudes and control profiles. Table 1 summarises the five attitudinal–control clusters within the REALISE Asia population named as follows: Well-adjusted and at least partly controlled (‘Well-adjusted’); In denial about symptoms (‘Rejectors’); Tolerating with poor control (‘Endurers’); Adrift and poorly controlled (‘Lost’); Worried with multiple symptoms (Worrier’) that are described in Table 1 (attitudinal and control characteristics) and Figure 2 (prevalence and control profiles). Further detail of the cluster analysis is provided in the online supplement.

To help realise the practical value of segmenting and characterising the REALISE Asia participants in terms of their asthma attitude–control profiles, we used a modified electronic Delphi (e-Delphi) procedure to generate consensus on asthma management recommendations tailored for each of the five clusters.

## Results

The 11 panel members comprised nine clinicians, one pharmacist and one Health Social Scientist working across seven countries—five in Asia (The Philippines, Malaysia, Singapore, Hong Kong and Korea) and two in Europe (UK and The Netherlands).

**Figure 3:**
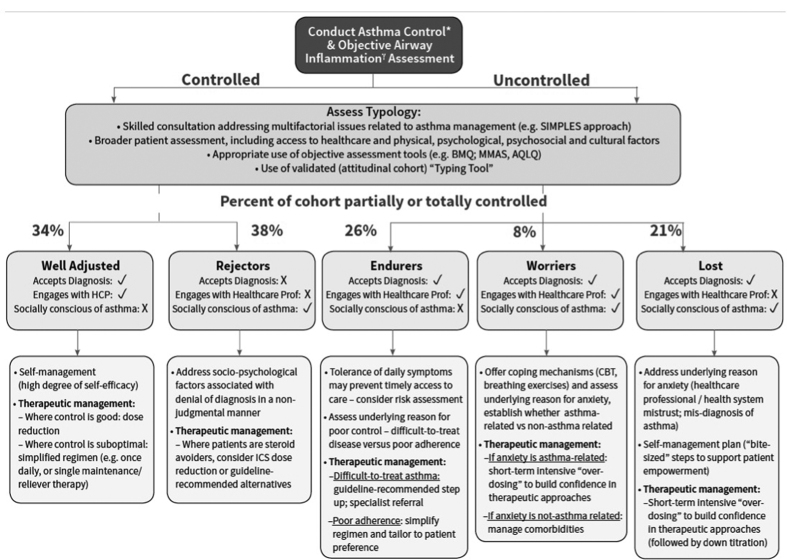
Management Pathway Algorithm for the attitudinal–control cluster consensus recommendations. *ACT, RCP3, GINA-based symptom control, SIMPLES; Yspirometry, peak flow, FeNO; ACT, asthma control test; ACQ, asthma control questionnaire; Acronyms, AQLQ, asthma quality of life questionnaire; BMQ. belief about medicines questionnaire; CBT, cognitive behavioural therapy; HADS, Hospital Anxiety and Depression Scale; MMAS, Morisky Medication Adherence Scale; RCP3, Royal College of Physicians; SIMPLES, Smoking, Inhaler technique. Monitoring, Pharmacology, Lifestyle, Education, Support.

Through Rounds 1 and 2, the panellists agreed that there was clinical utility in identifying all five attitudinal clusters as a way to tailor asthma management approaches. On completion of Round 3, consensus recommendations (those achieving the support of >80% of the panel) were identified for all attitudinal–control clusters. The consensus recommendations for identifying and managing the different attitudinal–control clusters are summarised in Table 2 and 3, respectively. Discussions as to how best to implement these recommendations resulted in a pathway illustration, Figure 3.

### Identification of Attitudinal–Control clusters

The consensus panel recommended approaches for assessing both control and attitude in the context of the patients’ medical and social circumstances.

Multiple options for asthma control assessment were suggested (Table 2), including: use of validated tests (e.g., Asthma Control Test, ACT; Asthma Control Questionnaire, ACQ; GINA-based evaluation of control status) and structured holistic assessments approaches (e.g., current smoking, inhaler technique; monitoring, pharmacology, education and support; SIMPLES^23^). To evaluate control, spirometry and peak flow were proposed and (if available) fractional exhaled nitric oxide (FeNO) assessment to detect airway inflammation. Panellists agreed that selection of the appropriate assessment approach should be guided by local healthcare resource and informed by cultural setting.

In-depth understanding of the clinical and social context was felt to be important in some patients. For example, assessment of ‘Lost’ patients should include evaluation of differential or co-morbid diagnoses (e.g., comorbidity and dysfunctional breathing assessments, potentially using lung function and FeNO testing).^24^ Similarly for patients classified as potential Worriers, the panellists recommended evaluation of patients’ global anxiety (e.g., HADs, dysfunctional breathing assessment) to assess whether the anxiety observed is specific to the patient’s asthma, or may be a more generalised behavioural trait.

**Figure 4:**
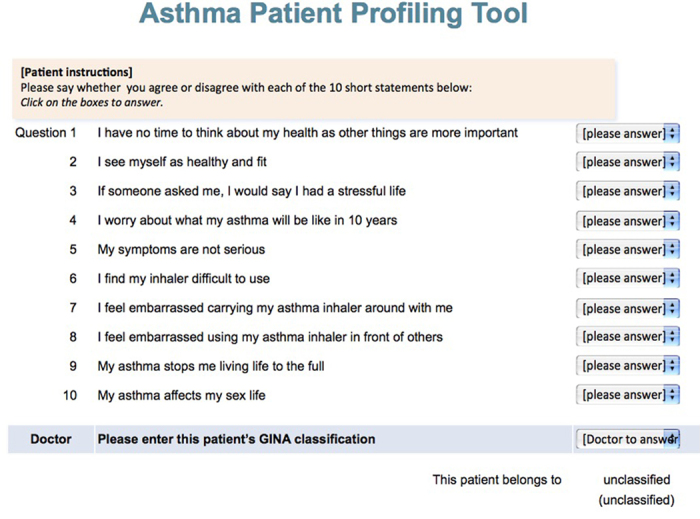
Attitudinal cohort profiling tool developed for use in clinical practice by the REALISE Asia investigators^22^. Patients must answer Agree/Disagree to each statement, 1–10. The patient's physician must indicate whether the patient has: controlled; partly controlled or uncontrolled asthma. Once complete, the patient's attitudinal classification is automatically generated.

Although there was widespread agreement among panel members that the clusters relate to clinically recognisable groups, it was felt that a typing tool (Figure 4) would have practical utility in ensuring accurate assessment and categorisation of patients, particularly in non-dedicated specialist centres.

### Pharmacological management

Optimisation of pharmacological management in line with GINA recommendations was a common theme across all clusters. However, panellists agreed there is no ‘one-size-fits-all’ approach to pharmacological management and that optimisation needs to be tailored to the attitudinal–control cohort, and at the individual patient level in line with their lifestyle requirements and preference (in keeping with the concept of personalised medicine^25^).

While optimised therapy in ‘Well-adjusted’ patients may involve dose-reduction or simplification of the treatment regimen (i.e., depending on the patient’s individual control status), pharmacological optimisation in ‘Rejectors’ must take into consideration the psychosocial determinants that result in those patients denying their diagnosis and failing to implement prescribed treatment. Concerns about steroid use might be addressed by reducing the dose of inhaled corticosteroid (ICS) to the minimum required to achieve symptom control or sometimes by use of ICS alternatives. Suitable treatment options for ‘Rejectors’ must also be pragmatic—recognising that these patients are likely to be intermittent treatment users (at best) and that therapy must optimise potential anti-inflammatory effects when used on this basis. In ‘Lost’ and ‘Worried’ patients, who have high levels of anxiety in relation to their asthma, a short-term use of higher-step therapy may be valuable to demonstrate the potential benefits of treatment in the short term before stepping down therapy to the lowest dose possible.

Optimisation of pharmacological management is of particular importance in ‘Endurers’ who tend to tolerate symptoms and to lack ambition (or awareness^26^) as to the level of control they should be able to achieve. Such behaviours make ‘Endurers’ a potentially at-risk group. Panellists felt the ‘Endurer’ attitudinal–control cluster comprises two clinically recognisable subgroups—those who are sub-optimally controlled owing to severe and/or difficult-to-treat asthma, and those whose sub-optimal control is due to poor implementation of their prescribed medication. The latter includes those unable to use their therapy optimally and those who do not use it as prescribed, either as a conscious choice or through a lack of awareness of the potential benefit of therapy. Where adherence appears to be the principal challenge to attainment of control, a simplified regimen and use of once-daily therapies may be alternative options.

Pharmacological interventions should be complemented by appropriate non-pharmacological approaches across all attitudinal–control clusters.

### Non-pharmacological approaches

The optimum approach for a given patient will be one that recognises the patient’s perception of their condition and that works in partnership with them, potentially including appropriate education and expert, non-judgmental consultation skills (including psychological approaches). In Asian communities the use of social networks and/or engagement of family and friends to help ‘normalise’ the patient’s perception of their condition may be of particular importance.

Competent consultation skills must be used to enable understanding of what lies beneath negative attitudes towards healthcare (e.g., ‘Lost’ and ‘Rejectors’) or at the root of patients’ anxieties and concerns (e.g., ‘Lost’ and ‘Worriers’). Part of optimising the patient–physician interaction is use of appropriate language to avoid potentially worrying or alienating patients. Terms such as ‘risk management plan’ should be avoided when speaking with anxious ‘Lost’ or ‘Worried’ patients. Instead, these patients would be reassured by use of empowering language and bite-sized management plans to build confidence. Phrases such as ‘asthma action plan’ may be better avoided when speaking with ‘Rejectors’ who tend not to accept their condition. Instead, the terms patients used to describe their condition and symptoms (e.g., ‘wheezing’ and/or ‘breathlessness’) should be adopted.

Self-management was considered to be important in all the clusters, but needs to be tailored to patients’ individual attitudes to their asthma. Although ‘Well-adjusted’ patients (who are most receptive to healthcare professionals’ recommendations and have the greatest sense of self efficacy) may need little support to take on the management of their asthma, those who are ‘Lost’ may require self-management plans detailing smaller-scale goals to permit regular attainment of targets to help build their confidence and may require regular follow-up after the initial consultation. ‘Endurers’, whose tolerance of their condition may result in a failure to respond in a timely and appropriate way to a worsening of symptoms, self-management plans with clear descriptions of symptom worsening and necessary actions may be valuable to support timely and appropriate access to healthcare.

## Discussion

### Main findings

This modified e-Delphi procedure illustrates the range of clinical management approaches required to tailor support for patients with different attitudinal–control profiles in routine care. Identifying patients’ attitudes is critical to successfully managing the complexities of asthma in the widely heterogeneous population that presents in routine daily care. The range of applicable potential management approaches can be narrowed by clustering patients with similar attitudes to healthcare and with similar control status, but optimum management still needs to be tailored to the individual determinants of each patient’s attitude and behaviour.

### Interpretation of findings in relation to previously published work

Patients’ poor perception of their level of asthma control within REALISE Asia echo similar findings from studies in Europe and North America.^14–20^ The subsequent clustering of patients by their attitudes towards healthcare and by their control status builds on prior work suggesting that asthma patients’ attitudes towards their medical professionals and treatment can be used to predict future risk of uncontrolled disease^27^ and that patient personality can influence adherence to asthma medication, control and health-related quality of life.^28^ The recommendations generated by this modified e-Delphi procedure seek to apply the recommendations of the 2014 GINA recommendations in terms of optimising asthma management and targeting treatment based on modifiable risk factors, patient preference and practical issues—optimisation of medication effectiveness by addressing inhaler technique and adherence.^29^

### Strengths and limitations of this study

The e-Delphi panel comprised Asian and European healthcare professionals and an Asian pharmacist and social scientist to ensure the recommendations recognised and reflected cultural and social nuances specific to the source population and care settings (e.g., 4 of the 8 countries that participated in REALISE Asia were represented in the panel), but also retained validity and potential applicability in other geographies and healthcare settings.

All eleven panellists contributed to all three of the mandatory elements of the modified e-Delphi process (i.e., the email-based interaction by which initial recommendations were sourced and subsequently voted for and prioritised). Partial involvement in the optional elements of the process (i.e., the elements that ‘modified’ the process from a standard Delphi) reflected the panellists’ prior experience of Delphi procedures (with Round 1.2 seeking to standardise the knowledge of all panellists) and panellists' geographical location (with the Round 2.2 meeting venue and time zone preferential to the majority Asian panellists). The overall retention of all panel members suggests there was broad agreement among panellists throughout the process, irrespective of their speciality discipline and geographical origin. Internal agreement was further strengthened by the iterative refinement of the recommendations through the consensus process.

The REALISE Asia recruitment criteria (i.e., asthma patients aged 18–50 years with physician-diagnosed asthma who had received at least two asthma prescriptions in the 2 preceding years and who had access to social media) may have resulted in some patients with intermittent asthma (or potentially misdiagnosed as having asthma) being included in the source population. However, all panellists felt the attitudinal–control clusters derived from the REALISE Asia study data were recognisable and relevant irrespective of the region, or the healthcare settings, in which they practiced. In addition, the panellists agreed that the distribution of patients between the cohorts may vary in different countries (see Supplementary Information for the distribution of cluster prevalence within the REALISE Asia participant countries). However, between-country differences were not felt to be a concern as societal attitudes towards chronic disease and healthcare interventions will vary between nations. Within that context, the recognition of the five clusters by physicians working in all eight participating countries offers confidence in the validity of their existence, both within Asia and likely beyond.

Although the attitudinal cohorts were defined to be distinct (i.e., mutually exclusive) as a result of their unique control status and attitude profiles, it was noted by panellists that some of the key characteristics (e.g., demonstration of health seeking behaviours) were common to more than one cluster (Table 1). The multifaceted cluster typing tool that was developed by the REALISE Asia investigators may assist in the accurate categorisation of patients.

The patient cluster identified by attitudinal–control cluster typing should not be considered to be an intrinsic patient characteristic. Patients' attitudes to healthcare can and do change over time as cognitive function varies (child to adult; adult to elderly) and levels of trust in healthcare alters as a result of patient experience and changing levels of care. Thus a patient may move from the ‘Lost’ cluster to the ‘Well-adjusted’ cluster as they gain more trust in healthcare. Indeed, moving towards the improved control profile of Well-adjusted patients should be management goals for the ‘Lost’, ‘Worrier’ and ‘Endurer’ clusters. Thus attitudinal–control classification should be assessed at regular intervals (e.g., at the time of annual asthma reviews) to ensure any transitions between categories can be identified and future interventions adjusted to optimise their relevance to the current disease state and mind-set of the patient.

In addition, there may be other patient clusters present in routine care (both in Asia and beyond) that were not represented in the REALISE Asia population (e.g., those without access to the internet and/or inactive on social media and so excluded from the source population). Indeed, it is acknowledged that the requirement for REALISE Asia participants to be adult asthma patients within with access to internet-based social media may reduce external validity of the clusters, or at least the relative prevalence of each. At the time of writing, there remain wide variations in access to the Internet and social media across Asia, although China is now the largest base of Internet users and, along with Japan, benefits from some of the fastest Internet speeds globally. Advances in Internet infrastructure, uptake and affordability in access in the region also see Malaysia, the Philippines, and Indonesia now being ranked among the top 15 countries globally in terms of Facebook users worldwide (with the Indonesia and the Philippines making the top 10 for Twitter).^30^

### Implications for future research, policy and practice

These recommendations formalise the traditional primary-care approach in which a patient with a medical condition is assessed and understood in the context of their clinical, psychological and social status.

The recommendations proposed by the panellists are intended to complement (not replace) pre-existing asthma recommendations. In the 2014 update, GINA recommended greater evaluation of patient risk and management of modifiable risk factors to complement (and guide) optimisation of step-wise pharmacological management. The panel’s recommendations support the GINA approach by providing guidance as to how pharmacological management approaches can be best tailored and optimised in routine care, including suggestions as to how best to engage and modify negative healthcare attitudes that can be a barrier to medication adherence and treatment outcomes. While the majority of the consensus management approaches are in line with GINA recommendations for all patients, some of the approaches are only specifically detailed for a subset of the five clusters. This reflects the panel’s view that these approaches were of particular relevance in the specified clusters, not that they would be inappropriate in a wider context (Table 3).

In Asian adult asthma patients, key modifiers of management approaches will include consideration of patients’ access to affordable high-quality healthcare, cultural attitudes and beliefs about medicines, literacy and health literacy, and a common dependence on support from family networks and social groups. Selection of appropriate objective assessment tools will be informed by the availability of local language translations and by whether they have been validated for use in a local/relevant population.

The potential risks of this management approach lie in too rigorous an application of the classification. Although a validated typing tool that allocates patients to one of the five clusters was felt useful to help direct management approaches, competent consultation skills remain key to determining the underlying drivers of an individual patients’ presenting characteristics was necessary to tailor those approaches to optimal effect.

These realities again highlight the critical role that close patient–physician interaction, good communication and a holistic approach to patient assessment hain capturing the nuances of an individual patient’s condition, the changes over time and the specific ways in which general management recommendations should be tailored at the individual level.

## Conclusion

The modified e-Delphi procedure successfully reached consensus recommendations from a multidisciplinary international panel of respiratory, pharmacology and health sciences experts working across a range of cultural, regional and healthcare settings. The recommendations offer a pragmatic approach to complement guideline-recommended management approaches. They aim to serve as an additional tool for guiding physicians in the optimum care of their patients with asthma. Their interpretation and application in clinical practice must be guided by thoughtful interpretation in the context of the individual patient’s experience and needs and changes over time. Further work is required to assess the utility and applicability of these complementary management recommendations for each patient cluster and across geographical and cultural regions.

## Materials and methods

### Modified e-Delphi procedure

The Delphi procedure is a structured research technique used to reach consensus via mail/email (Delphi/e-Delphi) on a specific topic among a panel of experts through feedback of information and iteration.^31,32^ The process is complete when consensus is reached.^33^ It has been used in several recent respiratory studies and research activities, including the International Primary Care Respiratory Group (IPCRG) as a structured approach to prioritisation of primary-care research needs.^34^

**Figure 5:**
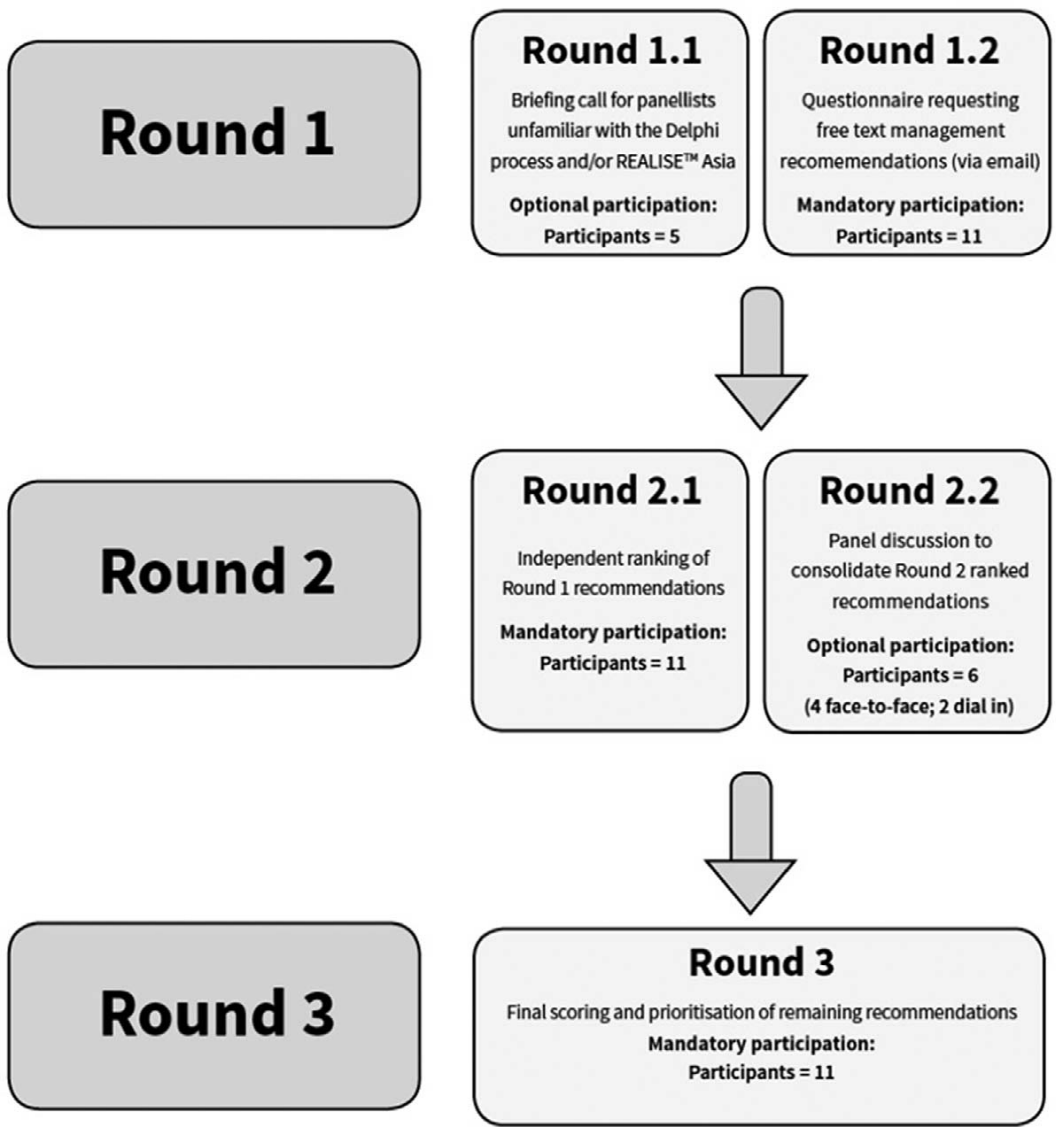
Modified e-Delphi procedure, summary of the mandatory (traditional) and optional (modified) rounds undertaken by the panellist and panellist participation at each step.

The modified Delphi procedure used to reach consensus within this study differed from the traditional procedure in that it incorporated two additional, complementary discussion steps (Figure 5).

### Panel selection

For this modified e-Delphi procedure, the Respiratory Effectiveness Group (REG; www.effectivenessevaluation.org) invited investigators from the REALISE Asia and REALISE Europe studies^13,20^ and a number of Asian and European representatives from the REG collaborator group.

### Consensus and feedback

The procedure consisted of five steps across three distinct rounds—Round 1: (i) circulation of briefing materials and a questionnaire requesting open, free text management recommendations, and (ii) participation in an optional complementary briefing call (NC, TTL, YVY, DP, ADW)); Round 2 required panellists to (i) review and priority scoring Round 1 recommendations, and (ii) participate in a panel discussion to consolidate recommendations (TTL, HP, DP, NC, CR, ADW)). The final round, Round 3, involved the final scoring and prioritisation of the remaining recommendations (Figure 5).

### Round 1 (March 2015)

All panellists received a letter explaining the rationale and context for the project and a written summary of key characteristics of the five attitudinal–control clusters identified within REALISE Asia. They were also sent a questionnaire asking them to indicate whether they believed identification of each cohort was clinically useful and invited to provide free text recommendations for each patient cluster to (a) identify patients with the attitude/control characteristics represented by that cluster, and for tailoring (b) pharmacological and (c) non-pharmacological approaches to managing these patients (Figure 5, Round 1.2; Supplementary Table 1a–e).

All panel members were also invited to join an optional teleconference to address any questions they had concerning the methodology and specifically to clarify the description of the clusters (Figure 5, Round 1.1; NC, TTL, YYV, DP joined).

### Round 2 (April 2015)

Round 2 involved two distinct steps—(i) independent scoring of Round 1 recommendations (via email), and (ii) sub-panel review, discussion and consolidation of recommendations (face-to-face meeting or virtual/telephone participation).

For the independent scoring phase (Figure 5, Round 2.1), each panel member received a collated summary of the free text recommendations collected during Round 1 (Supplementary Table 2a–c). For each cluster, panellists were requested to (i) indicate their agreement/disagreement with each recommendation and (ii) highlight and score the top three recommendations within each recommendation subset (identification, pharmacological and non-pharmacological recommendations).

Once the independent scoring phase was complete, the research coordinator collated and combined all scores for group review and discussion (Supplementary Table 3a–e). Panellists attending the VIII World Asthma Allergy and COPD Forum in Singapore (NC, DP, ADW, TTL) met face-to-face to review and discuss the ranked recommendations with a view to reducing the number of individual items and to work towards consensus (Figure 5, Round 2.2). Panellists not attending the Forum were invited to join remotely via teleconference (CR, HP joined). During the sub-panel discussions, similar recommendations were combined under a wider, more encompassing generic heading with the more specific original recommendations listed as examples beneath. In addition, recommendations that failed to receive majority endorsement (defined as endorsement/support from more than half of the panel, i.e., ≥6 of 11 possible votes) were removed.

### Round 3 (May–July 2015)

Final consensus was reached by circulating the consolidated Round 2 recommendations to all panellists and inviting them to vote for, and prioritise, the remaining recommendations for each cluster. Final consensus was defined as recommendations receiving Round 3 votes by >50% of panel members and the highest priority recommendations were those receiving the highest total priority score.

## Figures and Tables

**Table 1:** Summary of REALISE Asia attitudinal–control clusters^22^

		*Cluster 1: Well-adjusted*	*Cluster 4: Rejectors*	*Cluster 3: Lost*	*Cluster 4: Endurers*	*Cluster 5: Worrier*
		*Well-adjusted and at least partly controlled*	*In Denial about symptoms*	*Adrift and poorly controlled*	*Tolerating with poor control*	*Worried with multiple symptoms’*
Prevalence^a^ *n* (%)		713 (29)	429 (18)	332 (14)	715 (29)	278 (11)
GINA control status						
Controlled		34%	38%	3%	3%	0%
Partially controlled		48%	48%	19%	23%	8%
Uncontrolled		18%	14%	79%	74%	92%

*Key characteristics*						
Level of asthma control		High	High	Low	Low	Lowest
Level of confidence in asthma		Highest	High	Moderate	Low	Lowest
Perceived severity of asthma		Mild/Less	Mild/Less	Moderate	Moderate	Severe
Frequency of seeking information about asthma		Low	Moderate	High	Moderate	High
Level of concern about their asthma		Low	Low	Moderate	Moderate	High
Socially conscious about asthma		Lowest	High	Highest	Moderate	High
Descriptive summary		Generally cope well with their asthma Asthma has minimal impact on their daily lives Happy to go along with doctor’s advice No problem using their inhaler, reflecting carefree attitude	Refuse to accept asthma label Yet to come to terms with emotional burden of living with asthma Deprioritise their health despite some concerns about their asthma High social consciousness about using inhaler	High level of stress and anxiety about their asthma Asthma has high impact on their daily lives Avoid thinking about their health High asthma information seeking frequency but do not know where to turn for answers	Accept their condition and that they do not have control over it High acceptance of condition means they do not allow asthma to have a major impact on their daily life Low level of confidence in managing their asthma Less interested in seeking information than other uncontrolled patient types	Asthma is a constant worry on their mind Accept their condition but live with a high level of stress and anxiety about their asthma Exhibit high asthma information seeking frequency due to their concerns

a Within REALISE Asia responder population.

**Table 2:** Recommended tools and approaches to identify the patient clusters in clinical practice

*Cluster identification approaches*	*Attitudinal–Control Cluster*				
	*Well-adjusted*	*Rejectors*	*Endurer*	*Lost*	*Worrier*
Validated typing tool	**✓**	**✓**	**✓**	**✓**	**✓**
Consultation skills	**✓**	**✓** Open, non-judgmental, non-paternalistic attitude to allow patients to share their misgivings/doubts/reasons for denial	**✓**	**✓**	**✓** Risk profile and understand concerns
Asthma control assessment (ACT, ACQ, RCP3, GINA-based symptom control assessment)	**✓**	**✓**	**✓**	**✓**	**✓**
Exacerbation history/risk assessment	**✓**	**✓**	**✓**	**✓**	**✓**
FeNO	**✓**	**×**	**×**	**✓**	**✓**
Assessment of access to healthcare	**✓**	**×**	**×**	**×**	**×**
Lung function testing (spirometry, peak flow)	**✓**	**✓**	**✓**	**✓**	**×**
Beliefs about Medicines Questionnaire (BMQ)	**×**	**✓**	**✓**	**✓**	**✓**
Morisky Medication Adherence Scale (MMAS)	**×**	**✓**	**✓**	**×**	**✓**
Quality of life assessment (AQLQ)	**×**	**✓**	**✓**	**✓**	**✓**
Assess medication-related adverse events	**×**	**×**	**✓**	**✓**	**×**
Dysfunctional breathing assessment	**×**	**×**	**×**	**✓**	**✓**
Hospital Anxiety and Depression Score (HADS)	**×**	**×**	**×**	**✓**	**✓**
Inhalation technique assessment	**×**	**✓**	**×**	**×**	**✓**
Induced sputum analysis	**✓**	**×**	**×**	**×**	**×**
Medication history review	**✓**	**×**	**×**	**×**	**×**
Assessment of patient's perceived self-efficacy	**×**	**×**	**✓**	**×**	**×**
Comorbidity Assessment	**×**	**×**	**×**	**✓**	**×**

**✓**, specifically recommended for this patient group by the expert panel.

**×**, not specifically recommended for this patient group by the expert panel.

**Table 3:** Consensus management recommendations, split by REALISE Asia attitudinal cohort including cluster-specific qualifiers

*Recommendations*^a^	*Attitudinal–Control Cluster*				
	*Well-adjusted*	*Rejectors*	*Endurer*	*Lost*	*Worrier*
Optimised GINA Step-wise Management	**✓** If well controlled: consider step down If control could be improved: consider simplified treatment regimen	**✓** As usage/adherence is likely intermittent: encourage a regular maintenance regimen (e.g., fixed dose combination ICS/LABA)	**✓** Taking into consideration specific approaches for optimising adherence and targeting inflammation throughout the airways (e.g., extra-fine particle ICS)	**✓** Take individual patient's attitudes and beliefs into consideration and consider aggressive management’ (i.e trial of higher-step therapy to demonstrate efficacy and build confidence)	**✓** Where symptoms are the result of uncontrolled asthma, consider: •simplified treatment regimen to help optimise adherence •‘aggressive management’ to demonstrate efficacy and build confidence (e.g., ICS/LABA±LAMA or±LTRA)
Reduced or Non-ICS alternatives	**×**	**✓** If steroid avoidance is identified as a barrier to ICS containing	**×**	**×**	**×**
Avoid therapies that may causes anxiety	**×**	**×**	**×**	**✓** e.g., excess bronchodilator use	**✓** Where symptom are not the result of uncontrolled asthma
Comorbidity Management	**×**	**×**	**×**	**×**	**✓** Where symptoms are not the result of uncontrolled asthma
Education, cluster-specific recommendations	**✓** •Trigger avoidance (e.g., lifestyle; domestic and occupational irritants) •Inhaler technique assessment and training •Benefits of Medication adherence	**✓** •Tailored to patient’s perception of their condition (i.e., they do not accept they have ‘asthma’) •Trigger avoidance (e.g., lifestyle; domestic and occupational irritants)	**✓** •Benefits of Medication adherence	**✓** Tailored to patient’s literacy, health literacy and personal circumstances	**✓** •Trigger avoidance (e.g., lifestyle; domestic and occupational irritants) •Benefits of Medication adherence
Self-management (to empower and build confidence)	**✓** Key cohort to decide on the best/lowest dose of ICS that keeps them symptom free	**×**	**✓** Self-selecting self-managers. Management approaches include: asthma risk plan and ‘innovative’ (e.g., digital) solutions	**✓** Using objective-monitoring tools to help patient’s perception of their ability to monitor and self-manage	**✓** Individualised symptom management and risk management plan
Risk management	**×**	**✓** Not labelled as ‘asthma management plan’ (as they do not accept they have asthma)	**✓** To ensure timely and appropriate access to healthcare	**×**	**✓**
Follow-up: remote or less intensive	**✓**	**✓**	**×**	**×**	**×**
Follow-up: more frequent	**×**	**×**	**×**	**×**	**✓**
Improve expectations of treatment	**×**	**×**	**✓**	**✓**	**✓**
Psychological approach	**×**	**✓** •Tailor management style to specific patient characteristics •Motivational interviewing •Non-judgmental consultation style •Possible referral to specialist or psychologist	**×**	**✓** •Address concerns and low confidence in healthcare	**✓** •Possible referral to specialist or psychologist
Social Support	**×**	**✓** •Engage support from family, friend Group support & shared experiences (to help with de-stigmatisation)	**×**	**✓** •Engage support from family, friend •Group support & shared experiences (to help with de-stigmatisation	**✓** •Engage support from family, friend •Group support & shared experiences (to help with de-stigmatisation
Evaluate/manage comorbidities	**×**	**×**	**×**	**✓**	**✓**
Breathing exercises	**×**	**×**	**×**	**×**	**✓** Where symptoms are not the result of uncontrolled asthma

**✓**, specifically recommended for this patient group by the expert panel.

**×**, not specifically recommended for this patient group by the expert panel.

a Some recommendations/recommendation categories are relevant across all attitudinal–control clusters. However, the table summarises those highlighted by the panel as deserving particular attention in specific patient subgroups/clusters.
