# Personalising care of adults with asthma from Asia: a modified e-Delphi consensus study to inform management tailored to attitude and control profiles

**DOI:** 10.1038/npjpcrm.2017.2

**Published:** 2017-03-23

**Authors:** Alison Chisholm, David B Price, Hilary Pinnock, Tze Lee Tan, Camilo Roa, Sang-Heon Cho, Aileen David-Wang, Gary Wong, Thys van der Molen, Dermot Ryan, Nina Castillo-Carandang, Yee Vern Yong, on behalf of the Respiratory Effectiveness Group

**Correction to:** *npj Primary Care Respiratory Medicine* (2017) **27**, 16089; doi:10.1038/npjpcrm.2016.89; published online 5 January 2017

‘Dephi’ in the article title should be ‘Delphi’.

A correction has been published and is appended to both the html and pdf versions of this paper.

The name of the study was misspelled in the title and has been corrected.

