# Supplementary material for: Personalising care of adults with asthma from Asia: a modified e-Dephi consensus study to inform management tailored to attitude and control profiles
Source: NPJ Prim Care Respir Med. 2017 Jan 5;27:16089–. doi: 10.1038/npjpcrm.2016.89 (PMC5215112; doi:10.1038/npjpcrm.2016.89)
Supplement: Supplementary Information [file npjpcrm201689-s1.doc]

**Supplementary Materials**

**REALISE^TM^ ASIA SURVEY**

The REcognise Asthma and LInk to Symptoms and Experience [REALISE™] Asia Study assessed patients’ perception of asthma control and their attitudes toward treatment. The study targeted an accessible, real-life adult Asian population (n=2,467). Eligible patients were randomly sampled from (self-reported) asthma patient panels in eight Asian countries/regions: The People’s Republic of China (30%), Hong Kong (8%), Indonesia (7%), Korea (20%), Malaysia (6%), Philippines (6%), Singapore (8%), and Taiwan (12%). They were aged 18–50 years, received a minimum of two prescriptions in pre­vious 2 years and had access to social media. The respondent sample size was based on factors, including popu­lation size and implementation cost, within 4%–8% error margin at 95% confidence level; participation emails were issued to individuals between December 2013 and March 2014. Patients were asked to confirm they had a physician diagnosis of asthma and were asked about their asthma symptoms, exacerbations and treatment type, views and perceptions of asthma control, attitudes toward asthma management, and sources of asthma information. Patients who were employed (and/or had relatives employed in) the healthcare, pharmaceutical, advertising, or market research industries were excluded, as were any patients who had taken part in healthcare-related market research in the past 3 months.

To identify and try to understand the gaps and disparities between physician and patient perceptions in terms of the impact of asthma (and the way the disease is managed) in Asia a second phase of the study involved a physician survey within the same Asian region, involving face-to-face and/or online surveys of clinicians involved in the management of patients with asthma. Physician participants (n=1074) were recruited via email invitations to clinicians working at country hospitals, clinic registries and participating in online physician panels. To be eligible for inclusion specialist physicians had to spend a minimum of 50% of their time directly managing patients (seeing a minimum of 50 patients with asthma per month, at least 60% of whom were aged ≥12 years; general practitioners had to see a minimum of 30 patients with asthma per month, at least 60% of whom were aged ≥12 years.

Control measures used in the REALISE Asia Survey:

- Patient-perceived: based on patient’s responses to the question ‘Would you consider your asthma to be well controlled’ (Y 89%/N 11%)
- Guideline-defined assessment: compared to GINA controlled (50%), partly controlled (32%) and uncontrolled (18%). GINA control was also calculated based on patient responses to a number of questions (see footnotes to table below) from the REALISE Asia report

**REALISE ASIA ATTITUDINAL CONTROL CLUSTER IDENTIFICATION**

The REALISE^TM^ Asia investigators used a two-step approach to segment questionnaire responses and characterise patients in terms of their attitudes to health care. Factor analysis was used to condense the 27 attitudinal variables captured by the REALISE^TM^ Asia questionnaire to a more manageable list size. A statistical algorithm was used to establish linkages between the attributes and the extent to which they were correlated within respondents. This enabled differentiation of truly related (compared with apparently related) attitudes and resulted in the creation of nine summary variables. Scalar data was then recoded to simple agree/disagree statements (from agree-disagree scales) to avoid distortion of the data by national cultures, e.g. the fact that respondents in the Philippines, Malaysia and Indonesia may be more likely to express an extreme position compared to someone in Hong Kong.

Using cluster analysis respondents were then segmented into groups based on their ratings against the nine summary factors. The objective of the cluster analysis was to segment patients into groups where factor differences within a group were minimized and factor differences between groups were maximised.

The cluster analysis resulted in identification of five clusters, each containing asthma patients similar in their attitudes and behaviours; each cluster formed a distinct segment): ‘Well-adjusted and at least partly controlled’; ‘In denial about symptoms’; ‘Tolerating with poor control’; ‘Adrift and poorly controlled’; ‘Worried with multiple symptoms’.

To assist practicing physicians accurately assign patients to their correct attitudinal-control cluster, a 10-question (agree/disagree questions) was developed for physician administration, and for use in conjunction with a GINA-defined control assessment (see Figure). The tool was found to be highly predictive, with 76.2% accuracy of classification within the REALISE Asia sample (exceeding the generally accepted standard of 75% accuracy).

Physicians participating in the REALISE Asia physician survey recognised the differing needs and priorities for the five patient clusters and 57% agreed that different attitudes towards asthma affect how they manage those patients.

**Figure 1. Distribution of attitudinal-control clusters across the eight countries included in the REALISE^TM^ Asia survey**

KR=Korea; MY=Malaysia; SG=Singapore; HK=Hong Kong; CN=China; TW=Taiwan; ID=Indonesia; PH=Philippines

**DELPHI ROUND 1**

**Table 1 (a)-(e) Round 1 Management Recommendations Questionnaire requesting free-text recommendations from panellists for all REALISE^TM^ Asia Altitudinal Patient Cohorts**

**Table 1(a) Round 1 Questionnaire for the Well-Adjusted cohort**

**Patients who are well adjusted to their asthma**

**Key characteristics of these patients are:**

- Generally cope well with their asthma
- Asthma has minimal impact on their daily lives (emotionally or functionally).
- Happy to go along with doctor’s advice
- No problem using their inhaler, reflecting a more carefree attitudes and lower stress levels.

| **Summary characteristics** | **Level of agreement** |
| --- | --- |
| Level of asthma control (GINA-defined criteria) | High |
| Level of confidence in managing asthma | Highest |
| Perceived severity of asthma | Mild/Less |
| Frequency of seeking information about asthma | Low |
| Level of concern about their asthma | Low |
| Socially conscious about asthma | Lowest |

**Unmet needs**

Well-adjusted patients:

- Still experience symptoms and exacerbations
- Have room for improvement in terms of asthma control, even though they are well-adjusted to their condition

| **Question 1.1. Do you think it important to identify this particular patient cluster?** |
| --- |
|  |

| **Question 1.2. What tools do you currently use (or might you like to use) to identify this patient cluster?** |
| --- |
|  |

| **Question 1.3. Are there any pharmacological approaches might you consider for this patient cluster in particular?** |
| --- |
|  |

| **Question 1.4. Are there any non-pharmacological approaches might you consider for this patient cluster in particular?** |
| --- |
|  |

| **Question 1.5. Are there any other interventions you might consider for this patient cluster?** |
| --- |
|  |

**Table 1(b) Round 1 Questionnaire for the Rejector cohort**

**Patients who reject / refuse to accept their asthma**

**Key characteristics:**

- Refuse to accept the asthma label
- Yet to come to terms with the emotional burden of living with asthma.
- Deprioritise their health despite some concerns about their asthma
- High social consciousness about using their inhaler
- Most independent in managing asthma
- Need support to be more comfortable with using inhalers in social settings and being prepared to use them when necessary

| **Summary characteristics** | **Level of agreement** |
| --- | --- |
| Level of asthma control (GINA-defined criteria) | High |
| Level of confidence in managing asthma | High |
| Perceived severity of asthma | Mild/Less |
| Frequency of seeking information about asthma | Moderate |
| Level of concern about their asthma | Low |
| Socially conscious about asthma | High |

**Unmet needs:**

Rejectors:

- Feel embarrassed about using their inhalers in public
- Have problems with their inhalers
- Are more anxious about asthma attacks
- Continue to experiences symptoms and exacerbations, despite a good overall control profile.

| **Question 2.1. Do you think it important to identify this particular patient cluster?** |
| --- |
|  |

| **Question 2.2. What tools do you currently use (or might you like to use) to identify this patient cluster?** |
| --- |
|  |

| **Question 2.3. Are there any pharmacological approaches might you consider for this patient cluster in particular?** |
| --- |
|  |

| **Question 2.4. Are there any non-pharmacological approaches might you consider for this patient cluster in particular?** |
| --- |
|  |

| **Question 2.5. Are there any other interventions you might consider for this patient cluster?** |
| --- |
|  |

**Table 1(c) Round 1 Questionnaire for the Lost cohort**

**Patients who are lost at managing their asthma**

**Key characteristics:**

- High level of stress and anxiety about their asthma
- Asthma has high impact on their daily lives (emotionally and functionally).
- Avoid thinking about their health
- High asthma information seeking frequency - has unanswered questions and do not know where to turn for answers.
- Highest tendency to have problems with asthma inhalers

| **Summary characteristics** | **Level of agreement** |
| --- | --- |
| Level of asthma control (GINA-defined criteria) | Low |
| Level of confidence in managing asthma | Moderate |
| Perceived severity of asthma | Moderate |
| Frequency of seeking information about asthma | High |
| Level of concern about their asthma | Moderate |
| Socially conscious about asthma | Highest |

**Unmet needs:**

Lost patients:

- Need to have their confidence in doctors and the healthcare system restored.
- Feel embarrassed about using their inhalers in a social context.
- Have serious problems with inhaler use.

| **Question 3.1. Do you think it important to identify this particular patient cluster?** |
| --- |
|  |

| **Question 3.2. What tools do you currently use (or might you like to use) to identify this patient cluster?** |
| --- |
|  |

| **Question 3.3. Are there any pharmacological approaches might you consider for this patient cluster in particular?** |
| --- |
|  |

| **Question 3.4. Are there any non-pharmacological approaches might you consider for this patient cluster in particular?** |
| --- |
|  |

| **Question 3.5. Are there any other interventions you might consider for this patient cluster?** |
| --- |
|  |

**Table 1(d) Round 1 Questionnaire for the Endurer cohort**

**Patients who endure and tolerate their asthma**

**Key characteristics:**

- Accept their condition and that they do not have control over it
- High acceptance of their condition means they do not allow asthma to have a major impact on their daily life
- Low level of confidence in managing their asthma
- Less interested in seeking information than other uncontrolled patient types
- Neither highly impacted in daily lives nor socially conscious about their asthma.
- To some extent, they transfer the burden and responsibility for asthma care to their doctor
- Have suboptimal control levels and are not very confident in managing their asthma

| **Summary characteristics** | **Level of agreement** |
| --- | --- |
| Level of asthma control (GINA-defined criteria) | Low |
| Level of confidence in managing asthma | Low |
| Perceived severity of asthma | Moderate |
| Frequency of seeking information about asthma | Moderate |
| Level of concern about their asthma | Moderate |
| Socially conscious about asthma | Moderate |

**Unmet needs:**

Endurers:

- Have low expectations of the level of asthma control that the might achieve.
- Are not confident in their ability to manage their own asthma.
- Are not actively engaged, or proactive, in their own asthma management

| **Question 4.1. Do you think it important to identify this particular patient cluster?** |
| --- |
|  |

| **Question 4.2. What tools do you currently use (or might you like to use) to identify this patient cluster?** |
| --- |
|  |

| **Question 4.3. Are there any pharmacological approaches might you consider for this patient cluster in particular?** |
| --- |
|  |

| **Question 4.4. Are there any non-pharmacological approaches might you consider for this patient cluster in particular?** |
| --- |
|  |

| **Question 4.5. Are there any other interventions you might consider for this patient cluster?** |
| --- |
|  |

**Table 1(e) Round 1 Questionnaire for the Worrier cohort**

**Patients who are constantly worried about their asthma**

**Key characteristics:**

- Constantly worried about their asthma
- Accept their asthma but live with a high level of stress and anxiety about their condition
- Exhibit high asthma information seeking frequency due to their concerns
- Asthma has a high impact, in terms of stress
- Have the lowest confidence in their asthma of all patient types
- Most likely (of all patients) to seek information about their asthma
- Have the highest percentage of uncontrolled asthma (GINA-defined)
- Need emotional support to help them cope better
- Need help to be more comfortable using inhalers in social settings
- Relevant information may help to ease their anxieties
- Need help to be more comfortable using inhalers in social settings

| **Summary characteristics** | **Level of agreement** |
| --- | --- |
| Level of asthma control (GINA-defined criteria) | Lowest |
| Level of confidence in managing asthma | Lowest |
| Perceived severity of asthma | Severe |
| Frequency of seeking information about asthma | High |
| Level of concern about their asthma | High |
| Socially conscious about asthma | High |

**Unmet needs:**

Worriers:

- Experience substantial anxiety and stress associated with their asthma
- Are keen to source healthcare advice
- Need to have their confidence in their ability to manage their asthma improved
- May need emotional support to ease their anxieties
- Are socially conscious about using their inhalers in public settings

| **Question 5.1. Do you think it important to identify this particular patient cluster?** |
| --- |
|  |

| **Question 5.2. What tools do you currently use (or might you like to use) to identify this patient cluster?** |
| --- |
|  |

| **Question 5.3. Are there any pharmacological approaches might you consider for this patient cluster in particular?** |
| --- |
|  |

| **Question 5.4. Are there any non-pharmacological approaches might you consider for this patient cluster in particular?** |
| --- |
|  |

| **Question 5.5. Are there any other interventions you might consider for this patient cluster?** |
| --- |
|  |

**Table 2. Round 1 Results: free text responses**

**Table 2a. Response, by attitudinal-control cohort, to the question: What tools do you currently use (or might you like to use) to identify this patient cluster?**

| **Well-adjusted**  (Well-adjusted and at least partly controlled) | **Rejectors** (In denial about symptoms) | **Endurer** (Tolerating poor control) | **Lost** (Lost and poorly controlled) | **Worrier** (Worried with multiple symptoms) |
| --- | --- | --- | --- | --- |
| - Lung function testing (spirometry, peak flow) - Consultation skills - Validated typing tool - Standard / stock questions that can be incorporated in to the consultation in order to identify key patient characteristics and attitudes - Assessment of access to healthcare - Asthma control assessment (ACT, ACQ, RCP3, GINA-based symptom control assessment) + exacerbation history/risk assessment - FeNO - Induced sputum analysis - Review of medication history | - Lung function testing (spirometry, peak flow) - Consultation skills - Validated typing tool - Open, non-judgmental, non-paternalistic attitude to allow patients to share their misgivings / doubts / reasons for denial - Beliefs about Medicines Questionnaire (BMQ) - Asthma control assessment (ACT, ACQ, RCP3, GINA-based symptom control assessment) + exacerbation history/risk assessment - Morisky Medication Adherence Scale (MMAS) - Inhalation technique assessment - Assessment of medication-related adverse events Quality of life assessment (AQLQ) | - Lung function testing (spirometry, peak flow) - Consultation skills - Validated typing tool - Beliefs about Medicines Questionnaire (BMQ) - Asthma control assessment (ACT, ACQ, RCP3, GINA-based symptom control assessment) + exacerbation history/risk assessment - Morisky Medication Adherence Scale (MMAS) - Quality of life assessment (AQLQ) - FeNO - Assessment of medication-related adverse events - Assessment of patient's perceived self efficacy | - Lung function testing (spirometry, peak flow) - Consultation skills - Validated typing tool - Beliefs about Medicines Questionnaire (BMQ)  Asthma control assessment (ACT, ACQ, RCP3, GINA-based symptom control assessment) + exacerbation history/risk assessment - FeNO - Dysfunctional Breathing Assessment - Depression and Anxiety  Comorbidity Assessment Quality of life assessment (AQLQ) - Morisky Medication Adherence Scale (MMAS) - Hospital Anxiety and Depression Score (HADS). - Assessment of medication-related adverse events | - Beliefs about Medicines Questionnaire (BMQ) - Asthma control assessment (ACT, ACQ, RCP3, GINA-based symptom control assessment) + exacerbation history/risk assessment - Consultation skills (to risk profile and understand concerns) - FeNO - Morisky Medication - Adherence Scale (MMAS) - Quality of life assessment (AQLQ) - Hospital Anxiety and Depression Score (HADS). - Dysfunctional Breathing Assessment - Validated typing tool - Check inhaler technique |

**Table 2b.** Responses, by attitudinal-control cohort, to the question: “Are there any pharmacological approaches might you might consider for this patient cluster in particular?”

| **Well-adjusted**  (Well-adjusted and at least partly controlled) | **Rejectors** (In denial about symptoms) | **Endurer** (Tolerating poor control) | **Lost** (Lost and poorly controlled) | **Worrier** (Worried with multiple symptoms) |
| --- | --- | --- | --- | --- |
| - Consider step down therapy - Reduce dose of maintenance ICS - Simplified treatment regimen | - Simple dosing regimen (once- or twice-daily) - Leukotreine receptor antagonists for milder disease - Formoterol-containing ICS/LABA - Single maintenance and reliever therapy (MART) | - GINA step-wise management (optimise and step-up until control is achieved) - ICS/LABA combination therapy - Single maintenance and reliever therapy (MART) - Once-daily regimens - Small particle formulations - Formoterol-containing combinations | - Optimise GINA step-wise treatment - Single maintenance and reliever therapy (MART) - Consider oral therapy: Leukotriene receptor antagonists - ICS/LABA combination therapy | - Aggressive management - Simple treatment regimen - Step-wise management until control is achieved - Once-daily regimens - Single maintenance and reliever therapy (MART) - ICS/LABA combination therapy ± add-on LTRA - ICS/LABA combination therapy ± add-on LAMA |

**Table 2c. Responses, by attitudinal-control cohort, to the question: “Are there any non-pharmacological approaches you might consider for this in particular**

| **Well-adjusted**  (Well-adjusted and at least partly controlled) | **Rejectors** (In denial about symptoms) | **Endurer** (Tolerating poor control) | **Lost** (Lost and poorly controlled) | **Worrier** (Worried with multiple symptoms) |
| --- | --- | --- | --- | --- |
| - Education about asthma triggers (e.g. environmental exposures, hygiene advice) - Breathing exercises - Self-management plan Patient incentives - Inhaler technique assessment & training - Simplified patient follow up | - Prescribing instructions tailored to patient's own symptom/disease description (e.g. for "wheeze", for "chest complaint"; not asthma) - Motivational interviews and discussions and positive reinforcement of "good" behaviours - Education - tailored to patient's perception of their condition - Tailor management approaches to patient's lifestyle - Tailor management approach to patient's perception of efficacy - Take a psychological approach to patient interaction and management - Refer patient to a psychologist - Breathing exercises - Group support & experience sharing (asthma clubs, support groups) - Engagement of family ± friends to provide additional support - Self-management plans (although not labelled as an "asthma action plan") - Education about asthma triggers (e.g. environmental exposures, hygiene advice) - Frequent follow up | - Consideration for at risk register - Family member engagement to support implementation of management approaches - Realistic goal setting Individualised self-management and risk plans and self-management education - Motivational interviewing and empowerment - Frequent follow up - Education focused on treatment adherence - Group support & experience sharing (asthma clubs, support groups) - Technology-based solutions (e.g. Apps and smart-phone based monitoring) | - Education about asthma triggers (e.g. environmental exposures, hygiene advice, smoking) - Engage social support - family, friendship group support - Breathing exercises - Structured plans (bite-sized information) to guide self management - Use of objective monitoring tools (e.g. peak flow meters) and thresholds (e.g. 30% drop in FEF) to guide self-management - Management of patients' anxieties - Strategies to build the patient's confidence in their healthcare professional - Inhaler technique assessment & training - Assess presence of comorbidities (e.g. allergic rhinitis, dysfunctional breathing, vocal chord dysfunction) - Self-management plan - Psychological approach to consultations - Specialist referral - Counselling and/or referral to a psychologist - Close follow up (home visits, web-based management, telehealth) - Evaluate health literacy (and tailor education as necessary) | - Individual risk plan - Discuss potential occupational factors / triggers - Frequent follow up - Optimise management of comorbidities - Engage social support - family, friendship to support management recommendations - Education: improve understanding of feasible levels of asthma control & assess patient's perception of efficacy - Psychological approach to management  Referral to a psychologist/psychiatrist - Specialist referral - Education: about asthma triggers (e.g. environmental exposures, hygiene advice, smoking) - Education: self-management and asthma action plans to build confidence in self-management - Psychological approach to consultations Specialist referral - Counselling and/or referral to a psychologist |

**Round 2 voting and priority scores, by attitudinal patient cohort.**

**Table 3(a) Round 2 results:** endorsement and prioritisation of Round 1 recommendations for “Well-adjusted” patients (“gold” = 1^st^ priority; “silver” = 2^nd^ priority; “bronze” = 3^rd^ priority)

**Table 3(b) Round 2 results:** **endorsement and prioritisation of Round 1 recommendations for “Rejector” patients**
(“gold” = 1^st^ priority; “silver” = 2^nd^ priority; “bronze” = 3^rd^ priority)

**Table 3(c) Round 2 results:** **endorsement and prioritisation of Round 1 recommendations for “Lost” patients**
(“gold” = 1^st^ priority; “silver” = 2^nd^ priority; “bronze” = 3^rd^ priority)

**Table 3(d) Round 2 results:** **endorsement and prioritisation of Round 1 recommendations for “Endurer” patients**
(“gold” = 1^st^ priority; “silver” = 2^nd^ priority; “bronze” = 3^rd^ priority)

**Table 3(e) Round 2 results:** **endorsement and prioritisation of Round 1 recommendations for “Worried” patients**
(“gold” = 1^st^ priority; “silver” = 2^nd^ priority; “bronze” = 3^rd^ priority)
